# What factors attract people to play romantic video games?

**DOI:** 10.1371/journal.pone.0231535

**Published:** 2020-04-16

**Authors:** Mayu Koike, Steve Loughnan, Sarah C. E. Stanton, Midori Ban

**Affiliations:** 1 Department of Psychology, University of Edinburgh, Edinburgh, Scotland, United Kingdom; 2 Department of Systems Innovation, Graduate School of Engineering Science, Osaka University, Osaka, Japan; Kyoto University, JAPAN

## Abstract

People in romantic relationships often benefit from improved mental and physical health and well-being. Today, these relationships can be recreated using virtual agents. For instance, some people anthropomorphize and fall in love with a virtual partner in a romantic video game. Although previous psychological research has examined anthropomorphized agents, it has neglected virtual romantic relationships. This study aims to examine the desire to play underlying playing romantic video games (RVGs). In Study 1, 43 Japanese participants completed a survey about their desire to play RVGs and their current romantic relationship status. The research revealed that a human-like voice and the use of touch were perceived as important factors in anthropomorphized relationships. In Study 2, an independent sample of 281 Japanese participants replicated the results of Study 1 regarding the importance of voice and touch in RVGs. Moreover, we found that a desire to develop social skills and alleviate negative emotions independently desire to play RVG use. As an important first step, these findings reveal several factors which might contribute to developing a romantic relationship with a virtual agent.

## Introduction

Developing romantic feelings toward a fictitious character is not an alien concept. Many of us have felt imaginary romance towards people in books, plays, and films. Computers allow these virtual characters to become interactive and responsive; machines can mimic some of the core characteristics of a human romantic relationship (e.g., conversation). Today, these virtual romantic relationships are available in romantic video games (RVGs). Although games differ, generally the player takes the role of the protagonist that explores and engages in romantic–but not necessarily sexual–relations with a virtual agent or agents. The extent to which RVGs mimic real romance can be remarkable. For example, when Japanese game company KONAMI released the male-oriented ‘Love Plus’ in Japan, many players reported falling in love with one of the virtual agents. RVGs offer virtual romantic relationships not only for men, but also for women. For example, ‘Tokimeki Memorial girl's side’ is an RVG targeting female players released by KONAMI, the same company that released Love Plus. The effect was so strong that some people worried RVGs could replace real-world romance. Some ‘Love Plus’ players reported preferring their virtual girlfriend to real women [1]. In 2014, RVGs in Japan were worth $130million per annum, and in 2016 a single, leading company earned $102million from RVGs alone. The dramatic success of RVGs has been attributed to their capacity to mimic human romantic relationships [2]. Currently, the popularity of RVGs is growing in English-proficient countries such as the US, UK, and within continental Europe [2]. In short, RVGs are an increasingly common part of the landscape for romance worldwide.

Despite the popularity of RVGs however, research into the psychology of them has been limited. By providing emotional support and social engagement, romantic relationships benefit our physical and mental health [3, 4]. Despite these benefits, finding a partner and falling in love may prove difficult due to anxiety towards real-life relationships [5] and high standards for a romantic partner [6]. RVGs could be a solution that fulfills romantic needs and provide similar benefits to romantic relationships, circumventing anxiety and high expectations. In short, RVGs could be growing in popularity because they fulfil the psychological needs of a relationship, and avoid some of the barriers that hinder forming a romantic relationship. As a starting point, the current work aims to reveal the factors that motivate people to play RVGs.

### Psychological characteristics

What kind of romantic relationship makes us happy? What is the desire for beginning a romantic relationship? To answer such questions, we must first observe that a satisfying relationship is typically one that is healthy. That is, when a relationship is considered satisfying, usually it will have a positive impact on our physical and mental health. It is well-established that married couples have better psychological well-being and live longer than singles [7]. Satisfaction with current relationship status is a strong predictor of well-being [8]. Some research suggests that well-adjusted married individuals have better ambulatory blood pressure and feel much more satisfaction with life compared to single individuals [9]. Importantly, relationship quality between single and married individuals should be compared because, if people feel that their relationship quality is low, then being single is seen as better than being a dissatisfied married individual [9]. In summary, a satisfying relationship can help to increase well-being.

Self-esteem plays an important role in forming romantic relationships. People who have high self-esteem find their romantic relationships more rewarding [10]. Unfortunately for people with low self-esteem, this can serve as a barrier to the formation of healthy relationships. People with lower self-esteem tend to overgeneralize and feel impending rejection when their partner offends them [11]. Also, breaking up often reduces a person’s level of self-esteem [12]. Regarding relationship formation, lower self-esteem individuals prioritize self-protection and are thus less motivated to romantically connect with others [13]. By contrast, individuals with higher self-esteem overestimate the chance of acceptance from a potential partner, leading to increased confidence [14]. Therefore, lower self-esteem individuals may struggle to start a new relationship. Given that RVGs offer a ready, reliable route to relationship formation with low to zero levels of offence or rejection. That is, romantic virtual characters offer no rejection for partnership and they promise to give players a guaranteed romantic situation; thus RVGs may particularly appeal to people with low self-esteem.

In addition to not feeling good about the self overall, experiencing loneliness has an important influence on relationship formation. A key factor causing loneliness–particularly for young people–is the lack of a romantic partner [15]. In fact, romantic relationship status directly influences romantic loneliness, and unfulfilled belongingness correlates with a fear of being single [15]. Thus, having a romantic partner can reduce the feeling of loneliness and improve the feeling of security. Given that RVG offer an immediately available relationship, they may particularly appeal to lonely individuals.

### Pressures in a real-life romance

RVGs may be particularly appealing when a person foresees difficulty in finding and maintaining a real relationship. For instance, online dating is a modern way to find not only casual but also ongoing relationships. However, it also contains several risks. For example, the experience of multiple rejections from prospective partners might elicit feelings of lower self-esteem and increased depression [16]. Further, because prospective partners exercise choice, investments of time and money do not guarantee relationship formation–the partner can always say no and walk away. By contrast, RVGs are designed to be enjoyable, allow participants to simply ‘reload’ or ‘restart’ if they make a romantic blunder, and contain paths via which every partner can be seduced. Thus, the return on investment of effort seems more certain in RVGs than real-life romance. In addition to being successfully pursuable, RVG partners are created to meet the idealized standards of players, both in terms of appearance and psychology. For example, RVG characters never have ‘a bad day’, never get stressed about work or life outside the relationship, and if they place demands on us we do not want, we can simply restart the game with a new partner. Research on human relationships shows us that when our romantic expectations are unfulfilled, we experience lower satisfaction, resulting in less commitment and investment towards the romantic partner [17]. For RVG players this seems less likely to happen; virtual agents are designed to meet high romantic expectations.

In short, RVGs may appeal to people seeking higher self-esteem and/or greater life satisfaction, but also to those who are concerned that there are too many barriers to finding someone in real life. Importantly, those who are seeking higher self-esteem and/or greater life satisfaction might perceive barriers to achieving it in real life, while such barriers are absent from RVGs.

Physical contact is an important element in romantic relationships. Regarding touch, it is a primitive and strong function that has many benefits. When people touch or are touched by others, we release serotonin increasing positive mood, and decrease cortisol production leading to less stress [18]. Touching reduces pain [19], and hugs increase relationship satisfaction [20]. A positive effect of hugs includes increased oxytocin [21], which decreases stress [22]. These positive effects naturally emerge in the real world; [23] show that experiencing difficulty in resolving conflicts within a couple is negatively correlated with the level of physical affection, and the number of physical interactions are positively associated with increased satisfaction. Similarly, marital or cohabiting romantic couples experiencing a high frequency of romantic kissing report increased relationship satisfaction and lower levels of stress [24]. To put it simply, physical contact appears to be an important and rewarding component of a relationship.

Verbal interactions are also important for romantic relationships. In contemporary society, technologies such as e-mail and text are frequently employed in romantic communication. These technologies, which support smooth communication within a couple, aid relationship satisfaction. For example, Skype use has been found to increase satisfaction in long-distance relationships [25]. In addition, people in long-distance relationships consider video and audio chats to be more consequential than paper- or digital-based letters—since they can receive more intimacy [26]. Thus, not only physical touch but also verbal interactions are important to forming a good and satisfying relationship.

### Game characteristics

Touch and verbal communication appear relatively easy to establish with a human romantic partner, while posing more of a challenge for RVGs. It is clear from RVG industry trends that the importance of tactile and verbal interactions is widely acknowledged by different companies [27,28]. The distinctive features of the Nintendo DS game ‘Love Plus’–one of the most popular RVGs–is that the players directly communicate with their virtual girlfriend and can communicate with her by verbal and tactile interactions (e.g., stroking the girl’s hair, cuddling and kissing her) through the device. For example, [28] highlighted the importance of touch with the ‘Love Press’. They employed a Nintendo Wii balance board which players could use as a massage tool for virtual female characters. Thus, tactile interactions are seemingly an important function for RVG players, potentially helping to create a more human-like romantic relationship with the virtual partner.

Voice interactions are also an important factor in creating romantic connections between players of RVGs and the characters. For example, ‘Love Plus’ players reported enjoying the verbal function because they were called their name by the character with a real voice [27]. Similarly, [29] surveyed 20s- and 30s- aged female RVG players, showing the importance of voice interactions of RVGs as a factor for their game use. In short, voice interactions also appear to be a key factor to enhance a player’s desire to play RVGs.

There are many romantic games available globally [2]. However, the psychological factors which motivate people to play these games have been broadly neglected. Additionally, the virtual characters’ features (e.g., tactile feedback)–although important to human relationships [30,31]–have similarly eluded research. The current study seeks to understand the psychological aspects and physical characteristics of the game which can motivate people to play.

### The current study

People have a strong desire to initiate and maintain romantic relationships. It is clear that romantic relationships are beneficial for mental and physical health, and that they can be a potent source of satisfaction and self-esteem in our daily lives. RVGs fulfill many of these needs in a virtual environment.

There has been no prior psychological research on the factors that motivate people to play RVGs. Based on prior related work, we expect that these games will appeal to people with low self-esteem, who feel lonely and dissatisfied, and who set high idealized standards for their partners. We also anticipate that people who view the game as able to fulfil their needs for tactile and verbal intimacy will be particularly attracted to RVGs. We conducted two exploratory studies to examine these desires to play them. This study is beneficial for both RVG developers and players to understand what types of RVG functions are important for players and what factors motivate us to play them. While romantic relationships with ‘human to human’ and ‘human to non-human’ entities are different, but there are some common elements that attract people to begin a romantic relationship with a virtual agent. Our results are the first step in the development of the understanding of the psychology of RVGs.

## Study 1 (pilot)

Despite a growing market for romantic video games (RVG), the desires that attract people to play these games remain unstudied. In this study, we examined a range of potential psychological and in-game characteristics which may have motivated people to play.

### Methods

#### Participants

In total, 43 people (18 male, 25 female) aged 19–23 (*M* = 19.7, *SD* = .83) participated in the study. Students registered for psychology lectures at Doshisha University, Kyoto, Japan were recruited in exchange for 500-yen ($4.5 USD). This study was approved by Doshisha University Psychology Research Ethics Committee. We received electronic written consent by form on the first pages of the Qualtrics online survey. To take part, participants must have consented on this form beforehand.

#### Procedure

Using a Qualtrics online survey, participants first completed basic demographic questions. Then, they watched a short video clip outlining ‘Love Plus,’ a famous RVG in Japan. Next, they completed questionnaires measuring loneliness, life satisfaction, and self-esteem. We also asked about the significances of tactile and human voice interactions in RVGs for them in the same questionnaire. On completion, participants were thanked and paid. Participants were asked to take the survey in a quiet and private space.

#### Materials

All questions are available online via the Open Science Framework (https://osf.io/apxdf/?view_only=df24381290c14718aa8bfa2a1dd0176e).

Demographic questions included gender, age, living situation (1 = living by themselves, 2 = living with his/her parents), participation in societies, employment, quantity of friends, any previous experience in playing RVGs. We additionally asked about their current romantic-relationship status and satisfaction with their current partner or with being single (e.g., unsatisfied with being single).

A loneliness scale was derived from [32], including three questions (e.g., “How often do you feel that you lack companionship?”) assessed by a three-point Likert scale (1 = Hardly Ever, 3 = Often). Cronbach’s Alpha for the current sample was .72.

A life satisfaction scale was proposed by [33], which comprises five statements (e.g., “In most ways my life is close to my ideal”) assessed by a seven-point Likert scale (1 = Strongly disagree, 7 = Strongly agree). Cronbach’s alpha for the current sample was .88.

The Rosenberg self-esteem scale [34] contains 10 items (e.g., On the whole, I am satisfied with myself) and was completed using a four-point Likert-type scale (1 = Strongly Disagree, 4 = Strongly Agree). Cronbach’s alpha for the current sample was .88.

A ‘Love Plus’ video was used as an example of an RVG. A short video of ‘Love Plus’, which is 2.5 minutes long, was retrieved from the KONAMI official channel https://www.youtube.com/watch?v=oiJoVvyNvbA. ‘Love Plus’ is an RVG targeting individuals who are attracted to feminine women; we asked female participants to watch the video clip and answer the following RVG related questions (e.g., to what extent voice and touch are important in virtual and romantic relationships) as if they had been male players.

Desire to play ‘Love Plus’ was measured by a seven-point Likert scale (1 = very low, 7 = very high). This question (“To what extent do you want to play this game?”) was asked after watching the short video of ‘Love Plus’.

The importance of touch and voice in real relationships as well as RVGs were assessed by a seven-point Likert scale (1 = not at all, 7 = very much). We examined the importance of tactile and voice interactions in virtual romantic relationships compared with real romantic relationships (e.g., “To what extent do you think tactile communication is important in romantic relationships?”).

## Results

A correlation analysis was conducted to determine which variables were correlated with a desire to play ‘Love Plus’. Both correlations and descriptive statistics for all participants for the main variables can be found in Table 1.

**Table 1:** Correlations and descriptive statistics.

	Desire to play	Voice (real life)	Touch (real life)	Voice (RVGs)	Touch (RVGs)	Loneliness	Satisfaction	Self-esteem	Unsatisfied to being single
Desire to play	1								
Voice (real life)	.12	1							
Touch (real life)	.05	.40**	1						
Voice (RVGs)	.13	.40**	.40**	1					
Touch (RVGs)	.10	.05	.40**	.17	1				
Loneliness	.15	-.06	.11	-.01	.05	1			
Life- satisfaction	-.02	.16	.40*	.13	.10	-.48**	1		
Self-esteem	-.02	.09	.15	-.09	.02	-.49**	.61**	1	
Unsatisfied with being single	-.20	-.19	-.31	.07	-.52**	-.07	.04	-.04	1
Descriptive statistics									
Mean	3.43	5.93	6.86	5.60	4.23	1.78	3.87	2.45	4.14
S.D.	1.76	0.77	1.06	1.22	1.77	0.57	1.37	0.50	1.55

*p<0.05, two-tailed.

**p < 0.01, two-tailed.

The pattern of correlations observed here show a number of interesting results. Most strikingly, none of the psychological or game variables suggested by prior work (e.g., romantic relationships, life satisfaction and loneliness studies) robustly correlated with desire to play. The closest to significance was loneliness r (40) = .15, in the anticipated direction; however, the non-significant result means that it did not support our anticipation. According to [35], lonely individuals anthropomorphized non-human objects more; thus, we hypothesized that loneliness would be associated with the desire to play RVGs as lonely individuals might seek connection with virtual romantic characters more than others. The emergence of significant correlations between self-esteem and life satisfaction (*r* = 0.61) and self-esteem and loneliness (*r* = -0.49) replicate well-known effects [36], suggesting that the study was taken seriously by participants. In short, our results indicate that desire to play RVGs is unrelated to the variables suggested by prior works findings (e.g., romantic relationships, life satisfaction and loneliness studies), which we expect correlates with the desire to play RVGs. Interestingly, an examination of the mean ratings of touch and voice reveals that both are important when considering RVGs. A single-sample t-test was conducted to determine whether people cared about touch and voice above average (e.g., scale mid-point). A tactile interaction in RVGs was rated significantly above (*M* = 4.23, *SD* = 1.77) the scale midpoint (3.5), *t* (42) = 2.71, *p* < .01. As with voice, voice interaction in RVGs was rated significantly above (*M* = 5.60, *SD* = 1.22) the scale midpoint (3.5), *t* (42) = 11.33, *p* < .01.

## Study 2

Study 1 failed to find robust significant relationships between desire to play and the variables we derived from prior work. In Study 2 we implemented a number of changes. One weakness of Study 1 was the small sample size and the reliance on asking female participants to imagine being male players. In Study 2 we substantially increased our sample and no longer asked people to imagine being other players. Next, we excluded the psychological variables such as self-esteem and life satisfaction which failed to show a relationship from Study 1. Loneliness and the desire to play RVGs was not significant in Study 1; however, it was close to significant. Therefore we anticipate that a larger sample size may show patterns that study 1 did not. Study 1 used Love Plus but Study 2 uses Koi Kyu-Bu for men. Love Plus is a famous RVG for the Nintendo DS that allows players to use unique functions such as tactile interactions by using a DS pen. There are no RVGs which have the same unique functions on the Nintendo DS for women. In order to control the quality of RVG functions between men and women, we changed the DS game (Love Plus) to a phone application RVG for men ‘Koi Kyu-Bu’. Finally, we introduced the idea that people might want to play RVGs for reasons more related to the anticipated direct benefits received from the game. Specifically, we examined anticipated benefits of RVGs (e.g., RVGs would make players feel happy, RVGs would develop players’ social skills, and RVGs would reduce players’ loneliness).

### Methods

#### Participants

In total, 281 people (175 male, 106 female) aged 19–25 (*M* = 20.3, *SD* = .96) participated in the study. Students registered for psychology lectures at Doshisha University, Kyoto, Japan were recruited in exchange for course credit. This study was approved by the University of Edinburgh PPLS Research Ethics Committee. We received electronic written consent by form on the first pages of the Qualtrics online survey. To take part, participants must have consented on this form beforehand.

#### Procedure

All the participants completed our survey online (Qualtrics) via phones, computers, or tablets as in Study 1. After providing demographics, they watched a short advertisement on RVGs (approximately 2mins) with audio. We prepared two videos for each sexual preference ‘*Koi Kyu-Bu*!’ for males (https://youtu.be/7Os5RKJTR-U) and ‘*Sanrio danshi’* for females (https://youtu.be/n6Pk-ElsDnQ), and answered questions on RVGs: desire for playing RVGs, the importance of tactile and voice interactions in real relationships and RVGs, and the anticipated benefits of RVGs. We prepared two types of RVG video stimuli; one aimed at heterosexual men and one aimed at heterosexual women. Finally, participants reported their levels of loneliness. On completion, participants were thanked and received course credit. Participants were asked to complete the survey in a quiet and private space.

#### Materials

All questions can be found on the Open Science Framework (https://osf.io/apxdf/?view_only=df24381290c14718aa8bfa2a1dd0176e).

Demographic questions include gender, age, living situation (1 = living by themselves, 2 = living with his/her parents), participation in societies, part-time jobs, quantity of friends, and playing experience in RVGs. We also assessed their satisfaction with their relationship or satisfaction with being single. Next, they answered questions regarding their desire to play RVGs, the importance of voice and touch communication in the real and virtual romantic relationships, their general recognition on RVGs, and loneliness.

Anticipated Benefits Scale contains total 13 questions and these questions were evaluated by a seven-point Likert scale (1 = not at all, 7 = very much). We prepared original questions to mainly examine recognition on diverse emotional benefits and social skills affected by RVGs generally. (e.g., “To what extent do you think RVGs reduce loneliness/ develop your social skills? / allow you to feel fantasy love? / make you feel content? / increase your confidence?/ make you feel secure?/ reduce your mental stress?”).

Stimuli from video clips of RVGs were used for examples of RVGs. We prepared a short video of an RVG for each sexual preference: ‘*Koi Kyu-Bu*!’ for males and ‘*Sanrio danshi’* for females. The clips were retrieved from the official game company channel on YouTube and approximately lasted less than two minutes.

The desire (“To what extent do you want to play RVGs?”) was measured by a 100 point-scale (1 = low desire, 100 = high desire).

A loneliness scale [33] was used on the same scale as in Study 1. Cronbach’s alpha for the current sample was .69.

The importance of touch and voice in real relationships and RVGs was used on the same scale as in Study 1.

## Results

To start, we conducted an exploratory factor analysis to determine the structure of our anticipated benefits scale. We used an Exploratory Factor Analysis (EFA) and thirteen questions related to reasons for playing RVGs were factor analyzed using maximum likelihood analysis with Promax rotation. The analysis yielded two factors with eigenvalues greater than 1 which explained a total of 51.74% of the variance. Factor 1 was labelled ‘positive affect enhancement’ reasons to play RVGs. This first factor explained 41.50% of the variance. The second factor derived was labelled ‘skills acquisition’ to play RVGs. The variance explained by this factor was 10.24%. The EFA result can be found in Table 2.

**Table 2:** Factor loading matrix with promax rotation for anticipated benefit scale items.

		Factor Loadings	
		1	2
To what extent do you think romantic video games make you feel happy?		**.86**	-.05
To what extent do you think romantic video games allow you to feel fantasy love?		**.81**	-.18
To what extent do you think romantic video games reduce your mental stress?		**.71**	.08
To what extent do you think romantic video games increase your confidence?		-.04	**.85**
To what extent do you think romantic video games teach you useful skills for real romance?		-.11	**.77**
To what extent do you think romantic video games develop your social skills?		-.04	**.71**
To what extent do you think romantic video games let you try different romantic partner from the real one?		-.10	.22
To what extent do you think romantic video games characters have perfect personality compared to real partner?		.57	-.20
To what extent do you think romantic video games make you feel content?		.63	.23
To what extent do you think romantic video games make you feel secure?		.22	.59
To what extent do you think romantic video games help you to recover from the experience of broken hurt?		.16	.46
To what extent do you think you feel jealousy towards a virtual partner in romantic video games if your real partner plays it?		-.09	.40
To what extent do you think romantic video games reduce loneliness?		.29	.40
	Eigenvalue	5.40	1.33
	% of total variance	41.50	10.24
	Total variance		51.74

Factor loading in bold type were considered for cluster interpretation.

The first factor comprised items measuring positive affect. The three items representing this factor were: the degrees that RVGs render respondents feeling happy, fantasy love, and reduced mental stress. The factor was thus named the ‘positive affect enhancement’. The second factor comprises beliefs that RVGs build skills, specifically: increased confidence, developing social skills, and teaching useful skills for a real romance. The components led to naming the second factor as ‘skill acquisition’. Both new scales showed good reliability: Cronbach’s *α* = .78 for positive affect and Cronbach’s *α* = .79 for skill acquisition.

Next, we computed the correlations between all variables of interest. Correlations and descriptive statistics for the main variables can be found in Table 3.

**Table 3:** Correlations and descriptive statistics for the variables of all participants in study 2.

	Desire to play RVGs	Voice (real life)	Touch (real life)	Voice (RVGs)	Touch (RVGs)	Loneliness	Skills Acquisition	Positive Affect Enhancement
Desire to play RVGs	1							
Voice (real life)	-.05	1						
Touch (real life)	-.03	.51**	1					
Voice (RVGs)	.29**	.28**	.18**	1				
Touch (RVGs)	.23**	.23**	.18**	.55**	1			
Loneliness	.13*	-.05	-.10	.07	.08	1		
Skills Acquisition	.28**	-.07	-.07	.16**	.21**	.01	1	
Positive Affect Enhancement	.37**	.06	.02	.31**	.24**	.10	.57**	1
Descriptive statistics								
Mean	23.70	5.94	5.88	5.02	4.11	1.63	2.91	3.95
S.D.	27.13	1.16	1.11	1.54	1.68	.49	1.20	1.34

*p<0.05, two-tailed.

**p < 0.01, two-tailed.

Testing correlation, the desire of playing RVG and loneliness *r* (279) = .129, *p* < .031, social skills *r* (279) = 283, *p* < .01, and positive affect enhancement *r* (279) = .365, *p* < .01 were all positively correlated. From the findings, it is clear that the more people believe in the benefits of RVGs, the more they would like to play them. Also, lonely individuals have a higher desire of playing RVGs. Interestingly, a positive correlation between the importance of touch in virtual romantic relationships and social skills *r* (279) = .210, *p* < .01, and positive affect enhancement r (279) = .244, *p* < .01 was found. Also, a positive correlation between the importance of voice in virtual romantic relationships and social skills *r* (279) = . 157, *p* < .01, and positive affect enhancement *r* (279) = .311, *p* < .01 were recognized. Therefore, more participants believe the voice and touch interaction with virtual characters are important, more they believe to enhance positive affect and improve social skills through playing RVGs. A positive correlation between the importance of voice in real relationships and virtual romantic relationships was found, *r* (279) = .279, *p* < .01. The same trend was recognized between the importance of touch in real relationships and virtual romantic relationships, *r* (279) = .176, *p* < .01. Therefore it is implied that participants value equally touch and voice interactions both in real relationships and virtual romantic relationships. Also, the desire for playing RVGs was correlated with both the importance of touch in virtual romantic relationships *r* (279) = .234, *p* < .01 and voice in virtual romantic relationships, *r* (279) = .285, *p*< .01, respectively. To examine the relative effects of these relationships on desire to play we conducted a linear regression.

Multiple regression analysis was used to test if voice and tactile interactions, loneliness, skills acquisition, and positive affect enhancement significantly predicted participants' desire of playing RVGs. The results of the regression indicated the two predictors explained a significant proportion of variance in participants’ desire of playing RVGs (*R*^*2*^ = .217, *F* (5,275) = 15.29, *p* < .01). It was found that voice interaction in RVGs significantly predicted the desire of playing RVGs (*β* = .208, *t* (280) = 3.13, *p* < .01,), as well as positive affect enhancement (*β* = .36, *t* (280) = 5.40, *p* < .01).

We have used two different types of RVGs that are suitable for both male and female players. In order to explore the gender difference [37], we separated the data for men and women and re-conducted the correlation analysis. Correlations and descriptive statistics for the variables of male and female participants can be found in Table 4.

**Table 4:** Correlations and descriptive statistics for the variables of male participants (below diagonal) and female participants (above diagonal) in study 2.

	Desire to play RVGs	Voice (real life)	Touch (real life)	Voice (RVGs)	Touch (RVGs)	Loneliness	Skills Acquisition	Positive Affect Enhancement
Desire to play RVGs	1	.05	.11	.29**	.14	.11	.28**	.41**
Voice (real life)	-.12	1	.22*	.34**	.24*	-.09	.06	.13
Touch (real life)	-.12	.68**	1	.09	.18	-.05	.06	-.01
Voice (RVGs)	.27**	.27**	.20**	1	.46**	.08	.19	.29**
Touch (RVGs)	.29**	.24**	.17*	.59**	1	.03	.34**	.31*
Loneliness	.15*	-.03	-.12	.07	.11	1	.00	.09
Skills Acquisition	.29**	-.15	-.13	.14	.14	.01	1	.63**
Positive Affect Enhancement	.33**	.03	.03	.31**	.20**	.11	.54**	1
Descriptive statistics								
Mean (Male)	21.78	5.99	5.82	4.80	3.99	1.64	2.90	3.87
Mean (Female)	28.87	5.87	5.97	5.39	4.29	1.60	4.29	2.93
S.D. (Male)	25.95	1.12	1.17	1.61	1.69	.51	1.25	1.37
S.D. (Female)	28.83	1.23	1.01	1.35	1.64	.47	1.38	1.13

*p<0.05, two-tailed.

**p < 0.01, two-tailed.

Testing the correlation for men, the desire of playing RVG and loneliness *r* (173) = .151, *p* < .047, social skills *r* (173) = 283, *p* < .01, positive affect enhancement *r* (173) = .334, *p* < .01, the importance of voice in virtual romantic relationships *r* (173) = 269, *p* < .01, and the importance of touch in virtual romantic relationships *r* (173) = 288, *p* < .01 were all positively correlated. From the findings, we found the same directions of all participant data, such as the more males believe in the benefits of RVGs, the more they would like to play RVGs. Thus males especially look for psychological/mental improvement through virtual romantic relationships interactions. Additionally, there was a positive correlation between the importance of voice in real relationships and virtual romantic relationships was found, *r* (173) = .271, *p* < .01, and between the importance of touch in real relationships and virtual romantic relationships, *r* (173) = .176, *p* < .022. In short, the pattern of results for men closely mirrors the total sample.

For women, desire to play RVGs was significantly correlated with social skills *r* (104) = 281, *p* < .01, positive affect enhancement *r* (104) = .406, *p* < .01, the importance of voice in virtual romantic relationships *r* (104) = 290, *p* < .01. Like male participants, there was a positive correlation between the importance of voice in real relationships and virtual romantic relationships, *r* (104) = .342, *p* < .01; therefore, females especially value voice interactions in real relationships and virtual romantic relationships. In short, many of the same effects emerge for women only as for the total sample.

The mean for the desire to play RVGs (M = 23.70, SD = 27.13) is slightly low in Study 2. We conducted the correlation analysis separately by dividing those who scored higher than average and those who scored lower than average to see if there are different correlations based on their mean desire to play. However, it is out of the scope of the paper, thus we added the results in the supplementary materials (https://osf.io/apxdf/?view_only=df24381290c14718aa8bfa2a1dd0176e).

## General discussion

In two studies, we examined why people seek to play RVGs. We explored the psychological factors and game characteristics (touch and voice interaction functions) of RVGs that may motivate gameplay. In addition, we examined the anticipated benefits of playing RVGs as a factor desiring people to play them.

The results showed significant correlations between the importance of voice and touch in real and virtual romantic relationships among two studies with independent samples; however, we did not find a robust effect of psychological factors (e.g., self-esteem and life satisfaction). Loneliness is the only psychological factor to correlate with the desire to play. We found a significant correlation between the created factors of benefits of playing RVGs (social skills and positive affect enhancement) and the desire of playing RVGs. In study 2, our results indicate that lonely individuals may have a greater desire to have romantic interactions with a virtual character.

Regarding touch, it is a primitive and strong function that has many benefits. Touching reduces pain [19], and hugs increase relationship satisfaction [20]. With regards to neuroendocrinological benefits, touch increases the hormone oxytocin which enhances social connection and reduces cortisol, the stress hormone [38]. It may be that people are sensitive to this role of touch, and when they feel it can be achieved in an RVG have increased desire to play.

With regards to the game characteristics of voice, the more players think that an RVG can provide quality voice interaction, the more motivated they are to play. Female participants commented that hearing a human-like voice is very important to enjoying RVGs [39]. In recent years, the vocation of voice acting has become very popular in Japan, having become similar in popularity to musical idols [40]. Voice acting jobs are now the most ideal jobs among female junior high school students [41] in Japan RVGs are developed partially with that in mind. These gender trends support our findings, that is a positive correlation between the importance of voice and the desire to play RVGs and positive affect enhancement. Interestingly, the desire of playing RVGs was negatively correlated with the voice interaction in real life relationship for people who have higher ratings of the desire of playing RVGs. This finding has raised an important point that they equally value the importance of voice in real and virtual relationships; however, the importance of voice in real relationships would reduce the desire of playing RVGs due to the imagination gap between real and fantasy. [42] states RVGs can transport the players to romantic fantasy worlds that are different from the un-ideal real world. Understanding this context, it is natural that Japanese players will often appreciate voice interaction in RVGs and view it positively, not only for the inclusion of real voices by voice actresses, but too for the high quality of graphics for characters that mark an important desire to play RVGs [39].

In Study 2, two key factors that motivate people to play RVGs were discovered. The first was anticipated positive affect (positive affect enhancement). It is unexpected that anticipating the game to make the players feel better predicts a greater desire to play. Indeed, our participants are likely correct: playing casual video games increases mood and reduces stress [43]. The second factor is less intuitive, but potentially very interesting: anticipated romantic skill development (skills acquisition). It appears that people are drawn to RVGs in part because they feel they will gain skills to help in real world romances. The idea that people play video games to develop skills has been previously suggested (e.g., [39]) but this is the first time it has been demonstrated in RVGs. We already know that playing strategic games can increase problem-solving abilities [44], and the current work points to additional, social benefits.

## Limitations and future directions

There are several important limitations to this work. We chose to recruit Japanese participants given the prevalence of RVG usage in Japan. Although this provides a good environment to test these ideas, whether the results of the current study would replicate in samples with less exposure to RVGs remains unknown. China–with a growing interest and consumption of RVGs [45]–may be one important culture for further research. Considering cultural difference, in western cultures too we may observe an increase in RVG production and player base. Therefore it would be important to consider playing RVGs in other nationality samples.

Regarding experimental procedure, we did not apply a suitable RVG for female participants in study 1. Thus, we fixed this issue in study 2 by using two different RVGs particularly for female and male players; however, study 1 would still be limited.

Our interest in these studies lies in understanding the factors that motivate people to play. Accordingly, the two studies were conducted using online questionnaires and participants did not play RVGs. Thus, while these studies shed light on why people may be drawn to try playing RVGs, they cannot tell us why they continue to play or whether they experience the benefits that motivate their playing.

In general, players seem to have a feeling and expectation of improving their social skills when playing RVGs. This finding allows us to understand what players expect to benefit by playing RVGs, but this does not mean however that RVGs are truly capable of improving their feelings or skills in real life. Future work should examine the benefits of playing RVGs and also examine whether the desire for positive affect enhancement, romantic skills acquisition, and the alleviation of loneliness serve to maintain RVG play as well as attract it.

## Conclusion

The current research grants us some insight into why people are attracted to RVGs. Our studies show that the more people believe in the benefits of playing RVGs, the more they would like to play the games. Lonely individuals have a higher desire to play RVGs. Also, tactile and voice interactions are important in both real and virtual romantic relationships. Equally importantly, it shows us that a range of psychological factors (satisfaction, self-esteem) which we might expect to be related to desire to play RVGs are, in fact, not associated with the desire of playing RVGs. People were attracted to play RVGs with the expectation of gaining benefits by playing rather than their individual psychological situation (satisfaction, self-esteem). There are a limited number of studies exploring the psychology behind romantic relationships with virtual agents, and it is significant that we have taken the first step into the cultivation of a new field within video game and relationship psychology.

## References

[1] Rani A. 2013. Retrieved from BBC news website: https://www.bbc.co.uk/news/magazine-24614830

[2] Marsh J, Ogura J. 2016. Retrieved from CNN website: https://edition.cnn.com/2016/11/21/asia/romance-gaming-japan/index.html

[3] Slatcher RB, Selcuk E. A social psychological perspective on the links between close relationships and health. Current directions in psychological science. 2017; 26(1): 16–21. 10.1177/0963721416667444 28367003 PMC5373007

[4] Uchino BN. Social support and health: a review of physiological processes potentially underlying links to disease outcomes. Journal of behavioral medicine. 2006; 29(4): 377–387. 10.1007/s10865-006-9056-5 16758315

[5] Joel S, MacDonald G, Shimotomai A. Conflicting pressures on romantic relationship commitment for anxiously attached individuals. Journal of personality. 2011; 79(1): 51–74. 10.1111/j.1467-6494.2010.00680.x 21223264

[6] Conroy-Beam D, Goetz CD, Buss DM. What predicts romantic relationship satisfaction and mate retention intensity: mate preference fulfillment or mate value discrepancies? Evolution and Human Behavior. 2016; 37(6). 440–448.

[7] Burman B, Margolin G. Analysis of the association between marital relationships and health problems: an interactional perspective. Psychological bulletin. 1992; 112(1): 39 10.1037/0033-2909.112.1.39 1529039

[8] Lehmann V, Tuinman MA, Braeken J, Vingerhoets AJ, Sanderman R, Hagedoorn M. Satisfaction with relationship status: Development of a new scale and the role in predicting well-being. Journal of happiness studies. 2015; 16(1): 169–184.

[9] Holt-Lunstad J, Birmingham W, Jones B. QIs there something unique about marriage? The relative impact of marital status, relationship quality, and network social support on ambulatory blood pressure and mental health. Annals of behavioral medicine. 2008; 35(2): 239–244. 10.1007/s12160-008-9018-y 18347896

[10] Erol RY, Orth U. Self-esteem and the quality of romantic relationships. European Psychologist. 2017.

[11] Murray SL, Rose P, Bellavia GM, Holmes JG, Kusche AG. When rejection stings: How self-esteem constrains relationship-enhancement processes. Journal of personality and social psychology. 2002; 83(3): 556 10.1037//0022-3514.83.3.556 12219854

[12] Luciano EC, Orth U. Transitions in romantic relationships and development of self-esteem. Journal of personality and Social Psychology. 2017; 112(2): 307 10.1037/pspp0000109 27379474

[13] Murray SL, Derrick JL, Leder S, Holmes JG. Balancing connectedness and self-protection goals in close relationships: A levels-of-processing perspective on risk regulation. Journal of Personality and Social Psychology.2008; 94(3): 429 10.1037/0022-3514.94.3.429 18284291

[14] Cameron JJ, Stinson DA, Gaetz R, Balchen S. Acceptance is in the eye of the beholder: Self-esteem and motivated perceptions of acceptance from the opposite sex. Journal of Personality and Social Psychology. 2010; 99(3): 513 10.1037/a0018558 20649372

[15] Adamczyk K. An investigation of loneliness and perceived social support among single and partnered young adults. Current Psychology. 2016; 35(4): 674–689. 10.1007/s12144-015-9337-7 27891044 PMC5104760

[16] Marateck J.2018. Retrieved from CNN website: https://edition.cnn.com/2018/05/29/health/online-dating-depression-study/index.html

[17] Vannier SA, O’Sullivan LF. Great expectations: Examining unmet romantic expectations and dating relationship outcomes using an investment model framework. Journal of Social and Personal Relationships. 2018; 35(8): 1045–1066.

[18] Field T, Hernandez-Reif M, Diego M. Schanberg S, Kuhn C. Cortisol decreases and serotonin and dopamine increase following massage therapy. International Journal of Neuroscience. 2005; 115(10): 1397–1413. 10.1080/00207450590956459 16162447

[19] Mancini F, Nash T, Iannetti GD, Haggard P. Pain relief by touch: a quantitative approach. PAIN^®^. 2014; 155(3): 635–642. 24361816 10.1016/j.pain.2013.12.024 PMC3988987

[20] Murphy ML, Janicki-Deverts D, Cohen S. Receiving a hug is associated with the attenuation of negative mood that occurs on days with interpersonal conflict. PloS one. 2018; 13(10): e0203522 10.1371/journal.pone.0203522 30281606 PMC6169869

[21] Light KC, Grewen KM, Amico JA. More frequent partner hugs and higher oxytocin levels are linked to lower blood pressure and heart rate in premenopausal women. Biological psychology. 2005; 69(1): 5–21. 10.1016/j.biopsycho.2004.11.002 15740822

[22] Morrison I. Keep calm and cuddle on: social touch as a stress buffer. Adaptive Human Behavior and Physiology. 2016; 2: 344–362.

[23] Gulledge AK, Gulledge MH, Stahmannn RF. Romantic physical affection types and relationship satisfaction. The American Journal of Family Therapy. 2003; 31(4): 233–242.

[24] Floyd K, Boren JP, Hannawa AF, Hesse C, McEwan B, Veksler AE. Kissing in marital and cohabiting relationships: Effects on blood lipids, stress, and relationship satisfaction. Western Journal of Communication. 2009; 73(2): 113–133.

[25] Hampton AJ, Rawlings J, Treger S, Sprecher S. Channels of computer-mediated communication and satisfaction in long-distance relationships. Interpersona: An International Journal on Personal Relationships. 2018; 11(2): 171–187.

[26] Janning M, Gao W, Snyder E. Constructing shared “space”: Meaningfulness in long-distance romantic relationship communication formats. Journal of Family Issues. 2018; 39(5): 1281–1303.

[27] Livedoor news. 2009. Retrieved from https://news.livedoor.com/article/detail/4358119/

[28] Yamashita T, Kato T, Yokota M, Yamamoto N, Shirai A. LovePress++: A new love serious game which responds to physical communication. Social Interaction. 2011.

[29] Trend Lab.2014: Retrieved from http://www.trendsoken.com/report/entertainment/722

[30] Simmering MJ, Fuller JB, Marler LE, Cox SS, Bennett RJ. Tactile interaction norms and positive workplace touch. Journal of Managerial Issues. 2013 132–153.

[31] Sehlstedt I, Ignell H, Backlund Wasling H, Ackerley R, Olausson H, Croy I. Gentle touch perception across the lifespan. Psychology and aging. 2016; 31(2): 176 10.1037/pag0000074 26950227

[32] Hughes ME, Waite LJ, Hawkley LC, Cacioppo JT. A short scale for measuring loneliness in large surveys: Results from two population-based studies. Research on aging. 2004; 26(6): 655–672. 10.1177/0164027504268574 18504506 PMC2394670

[33] Diener ED, Emmons RA, Larsen RJ, Griffin S. The satisfaction with life scale. Journal of personality assessment. 1985; 49(1): 71–75. 10.1207/s15327752jpa4901_13 16367493

[34] Rosenberg M. Society and the adolescent self-image. Princeton, NJ: Princeton University Press 1965.

[35] Epley N., Waytz A., Akalis S., & Cacioppo J. T. When we need a human: Motivational determinants of anthropomorphism. Social cognition. 2008; 26(2): 143–155.

[36] Bozoglan B, Demirer V, Sahin I. Loneliness, self-esteem, and life satisfaction as predictors of Internet addiction: A cross-sectional study among Turkish university students. Scandinavian Journal of Psychology. 2013; 54(4): 313–319 10.1111/sjop.12049 23577670

[37] Dong G., Wang L., Du X., & Potenza M. N. Gender-related differences in neural responses to gaming cues before and after gaming: implications for gender-specific vulnerabilities to Internet gaming disorder. Social cognitive and affective neuroscience. 2018; 13(11), 1203–1214. 10.1093/scan/nsy084 30272247 PMC6234325

[38] Ellingsen DM, Leknes S, Løseth G, Wessberg J, Olausson H. The neurobiology shaping affective touch: expectation, motivation, and meaning in the multisensory context. Frontiers in psychology. 2016; 6: 1986 10.3389/fpsyg.2015.01986 26779092 PMC4701942

[39] Iguchi T. An investigation into video game use by university students with a particular focus on game genre and motivation. Journal of information and communication research. 2015; 33(2).

[40] Rogers K. 2015. Retrieved from Japan Today website: https://japantoday.com/category/features/an-inside-look-at-a-tokyo-voice-acting-academy

[41] Sony Life planner value. 2017. Retrieved from Sony life website: https://www.sonylife.co.jp/company/news/29/nr_170425.html

[42] Taylor E. Dating-simulation games: Leisure and gaming of Japanese youth culture. Southeast Review of Asian Studies. 2007; 29: 192–208.

[43] Russoniello CV, O'Brien K, Parks JM. The effectiveness of casual video games in improving mood and decreasing stress. Journal of CyberTherapy and Rehabilitation. 2009; 2(1).

[44] Adachi PJ, Willoughby T. More than just fun and games: The longitudinal relationships between strategic video games, self-reported problem solving skills, and academic grades. Journal of youth and adolescence. 2013; 42(7): 1041–1052. 10.1007/s10964-013-9913-9 23344653

[45] Pettman D. Love in the Time of Tamagotchi. Theory, Culture & Society. 2009; 26(2–3): 189–208.

